# Immunoglobulin gene analysis as a tool for investigating human immune responses

**DOI:** 10.1111/imr.12659

**Published:** 2018-06-26

**Authors:** Deborah Dunn‐Walters, Catherine Townsend, Emma Sinclair, Alex Stewart

**Affiliations:** ^1^ Faculty of Health and Medical Sciences University of Surrey Guildford UK

**Keywords:** antibody, B cell, human, repertoire

## Abstract

The human immunoglobulin repertoire is a hugely diverse set of sequences that are formed by processes of gene rearrangement, heavy and light chain gene assortment, class switching and somatic hypermutation. Early B cell development produces diverse IgM and IgD B cell receptors on the B cell surface, resulting in a repertoire that can bind many foreign antigens but which has had self‐reactive B cells removed. Later antigen‐dependent development processes adjust the antigen affinity of the receptor by somatic hypermutation. The effector mechanism of the antibody is also adjusted, by switching the class of the antibody from IgM to one of seven other classes depending on the required function. There are many instances in human biology where positive and negative selection forces can act to shape the immunoglobulin repertoire and therefore repertoire analysis can provide useful information on infection control, vaccination efficacy, autoimmune diseases, and cancer. It can also be used to identify antigen‐specific sequences that may be of use in therapeutics. The juxtaposition of lymphocyte development and numerical evaluation of immune repertoires has resulted in the growth of a new sub‐speciality in immunology where immunologists and computer scientists/physicists collaborate to assess immune repertoires and develop models of immune action.

## 1 INTRODUCTION

The unique character of adaptive immune receptor genes has been exploited in numerous ways to investigate the human immune system. Knowledge of lymphocyte development processes, and inferences based on existing paradigms of immune mechanisms, enable us to use the unique information embedded in the DNA sequence of the immune receptor repertoires to study human immune responses, where previously such insights could only be gained in animal models. In particular, B cell receptors (BCR) offer a wealth of information, being subjected to somatic processes of mutation and class switching after activation by antigen. Since these receptors can be secreted as antibodies they are of interest in many different areas of immunology as well as in the pharmaceutical industry where there are already more than 50 therapeutic antibodies approved for clinical use with many more in the pipeline.^1^ In addition, the elucidation of BCR specificities facilitates their use as single chain fragment variable regions (ScFv) in making Chimeric antigen receptors for T cell immunotherapy (CAR‐T cells).^2^

The clonal selection theory of immune responses is predicated on the existence of a hugely diverse set of specificities, from which the chance of finding a match to the antigen is high. Cells that respond to antigen are expanded in the repertoire, may also be affinity matured in the germinal center, and are therefore able to meet the challenge in force across many different anatomical sites. Resolution of the response after the infection is defeated leaves behind memory cells carrying the effective BCRs in order to provide faster and more efficient protection, with greater affinity, should the same challenge be encountered again. The potential diversity of the naïve immunoglobulin repertoire has been estimated to be in excess of 10^18^, which is 10^5^ times more than the estimated number of B cells in the body.^3^ The enormous diversity facilitated by V(D)J recombination has the disadvantage that some B cells may carry receptors that bind self‐epitopes, leading to autoimmune disease, so we need mechanisms of tolerance to remove such cells. B cell receptors which bind self‐antigen in the bone marrow are selected against via receptor editing (where the light chain of the B cell receptor is exchanged for a different light chain in an attempt to avoid self‐reactivity) or cell death. B cell receptors which do not bind self‐antigen proliferate and are released into the peripheral blood. Autoimmune disease may occur when central tolerance fails to remove autoreactive B cells before they leave the bone marrow. Several autoimmune diseases are associated with defective central tolerance mechanisms, for example, systemic lupus erythematosus (SLE),^4^ rheumatoid arthritis (RA)^5^, and type 1 diabetes.^6^ Autoimmune disease can also be a result of failed peripheral tolerance mechanisms, where self‐reactivity is acquired outside the bone marrow and needs to be removed. The affinity maturation process of adapting to immunological challenge may, in itself, create autoreactive specificities which require removal from the repertoire.^7^ In our own work, we have exploited the unique nature of immunoglobulin gene generation and maturation to investigate B cell dissemination and development in humans, especially with regard to how B cell protection diminishes, and autoimmune risk increases, with age.^8^ Along this journey, we find that repertoire analysis methods also provide information about intrinsic processes of immunoglobulin diversity generation that may be of benefit in therapeutic antibody design and discovery.

## 2 GENERATION OF B CELL DIVERSITY

Immunoglobulin genes are initially formed by gene rearrangement processes during B cell development in the bone marrow. Upon antigen activation they undergo further diversification by processes of somatic hypermutation and class switching in the periphery.

### 2.1 Gene rearrangement

**Figure 1:**
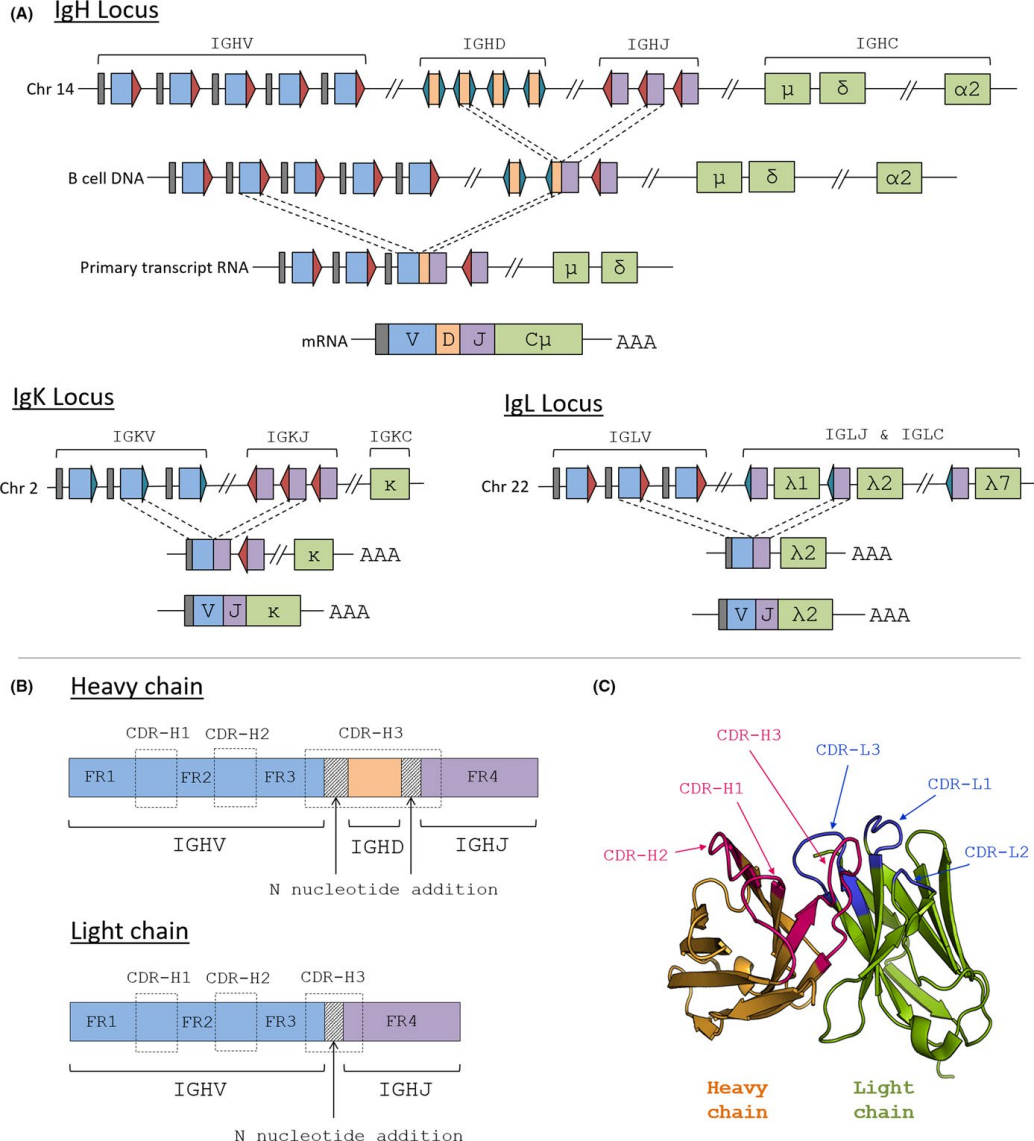
(a) Variable (V), Diversity (D) and Joining (J) gene segments are arranged in a non‐functional state in the germline. During V(D)J recombination, a V, a D and a J gene segment (just V and J in the case of light chains) are brought together at random. RSS sequences ensure gene segments are recombined in the correct order to form a functional variable region sequence. Blue, orange and purple rectangles represent V, D, and J gene segments, respectively, with gray leader regions upstream of the V genes. Turquoise and red triangles represent 12RSS and 23RSS, respectively. Constant region exons are represented by green rectangles. (b) Functional variable regions are composed of four conserved structural framework regions (FR) and three more diverse complementarity determining regions (CDR). The CDR3 regions are the most diverse as they span multiple gene segments and contain random nucleotide addition. C) The CDR loops make the most contact with antigen (PDB ID: 1FVC)

B cell diversity is achieved initially by rearrangement of Variable (V), Diversity (D) and Joining (J) immunoglobulin genes; VDJ for heavy chains and VJ for light chains (Figure 1a). The mechanism for gene rearrangements involves the use of recombination activating genes (RAG1 and RAG2) which recognize recombination signal sequences flanking the V, D, and J genes.^9^, ^10^ There are three different loci for the genes involved in VDJ recombination: on Chromosome 14 for the heavy chain genes *IGHV IGHD IGHJ*, on chromosome 2 for the *IGKV* and *IGKJ* kappa light chain genes and chromosome 22 for the *IGLV* and *IGLJ* lambda light chain genes.^11^ Each BCR comprises two identical heavy chains and two identical light chains, and the sites of the BCR most in contact with antigen are known as complementarity determining regions (CDRs). In the Fragment variable (Fv) part of the BCR, encoded by V(D)J regions, there are three CDRs interspersed between four framework regions (Figure 1b and c). CDRs 1 and 2 are encoded within the *IGHV/IGKV/IGLV* genes and therefore the variability in CDR1 and 2 in the repertoire is correlated with *IGV* gene usage. The CDR3 regions are the most variable, as they are encoded by the regions of the immunoglobulin where the different gene segments join together. Since light chain rearrangement involves only V and J regions, the CDR‐L3 is less diverse than the CDR‐H3, where the heavy chain region involves two different joining sites, between IGHV‐IGHD and between IGHD‐IGHJ as well as the *IGHD* genes. Diversity at these joining sites is increased in the CDR3 regions because the processes of gene rearrangement are imprecise, exonucleases may remove nucleotides and nucleotides are randomly added in the process by the enzyme Terminal deoxynucleotidyl Transferase (TdT). Only B cells will have a rearranged immunoglobulin gene and this has been quite an advantage working with limited availability of human tissue, as cell purification prior to any PCR is not necessary. Indeed, Ig gene analysis has been used to establish the presence of B cells in a tissue, for example, the presence of B cells in the human thymus.^12^

### 2.2 Hypermutation

Unlike T cells, B cells can further diversify during an active immune response by somatic hypermutation,^13^ a process which requires activation induced cytidine deaminase (AID)^14^ and additional help, such as from T follicular helper cell interactions.^15^ Somatic hypermutation takes place predominantly in the germinal center of follicles, where a Darwinian process of expansion, mutation and selection occurs, known as affinity maturation.^16^, ^17^ Cells acquire just one or two Ig variable region mutations in between rounds of selection^18^ and maturing cells exit the process as memory or plasma cells.^19^ Hence, when looking at the immunoglobulin gene rearrangements in a sample, the presence of mutations, in comparison to germline sequences, makes it evident that the cell has been activated by antigen. Thus, we could show for the first time that even though the B cells of the splenic marginal zone were not class switched, retaining IgM functionality, they were still antigen‐experienced cells as their Ig genes were mutated.^20^ In chronic lymphocytic leukemia (CLL) the extent of mutation was investigated to try and understand the etiology of the disease and it was found that there were two different classes of CLL with prognostic significance, those with mutated immunoglobulin genes and those carrying germline immunoglobulin genes.^21^ The extent of hypermutation may reflect the ongoing activation of a B cell clone and, in agreement with this, we have found that the mucosal barrier environment, where there is constant immune challenge, holds B cells and plasma cells with highly mutated Ig genes compared to systemic tissues.^22^, ^23^, ^24^ The extent of hypermutation has also been used to infer the likely activation pathway of a repertoire, with the assumption being that a T‐dependent response would always produce B cells carrying more highly mutated Ig genes than a T‐independent response. There is some evidence for this since patients with CD40L deficiency, whose B cells are unable to receive traditional T cell help, have fewer mutations in their class switched repertoire than controls.^25^ Therefore, a study of the human immune response to Dengue infection, which showed a hypomutated repertoire, lead to a model of Dengue immune response involving the T‐independent repertoire as well as the T‐dependent response.^26^

The question of whether an antibody has undergone antigen selection as part of its development has been asked in the context of studies on vaccine development, infectious disease, lymphomas and leukemias and autoimmune diseases. The initial hypothesis was that statistical comparison of replacement and silent mutation distribution across the IGHV gene would differ in an antigen‐selected gene compared to the mutation expected if it were completely random with no selection pressure. Such that an antigen‐selected gene would have more replacements than silent mutations in the CDRs which encode the antibody binding site, and conversely more silent than replacement mutations in the framework region of the antibody that is needed for antibody structural integrity.^27^ Calculations then had to be modified to account for our discovery that even in the absence of selection, in out‐of‐frame gene rearrangements there were more mutations in CDRs than framework regions.^28^ With the later determination of mutational hotspots,^29^, ^30^ that are the result of AID targeting and other DNA repair biases,^31^, ^32^ incorporation of targeting data into more complex algorithms enable improved prediction of whether a repertoire of antibodies has been selected or not.^33^ Other nuances, such as positional effects with respect to transcription initiation sites,^34^ intrinsic codon bias toward those more susceptible to amino acid change in CDRs^35^ or individual codon mapping across the repertoire,^36^ can also be taken into consideration. Analysis of hypermutation in the context of gene families, where the evolution of a B cell clone can be mapped by a phylogenetic study of hypermutation, can provide further insights and inferences to understand B cell biology (see Section 4.3 below).

### 2.3 Class switching

The function of an antibody can be varied by changing its Fc (Fragment constant) region, while retaining the specificities encoded and matured in the V(D)J arrangements of the Fv region, so when taking inference from a study of repertoires, in order to understand the biology of an immune response, it is important to know what kind of receptor is being studied. Naïve B cells have IgM and IgD on their surface and they may develop into plasma cells secreting IgM or they may undergo class switching to a different isotype. Secreted IgM may not have been through affinity maturation, but the avidity of the molecule may be quite high due to the ability of IgM to form pentameric molecules with 10 binding sites. Pentameric IgM can therefore form an ideal shape for complement activation and also facilitate the formation of antigen‐antibody immune complexes to be better recognized by other components of the immune system. The large size of pentameric IgM means that it cannot readily pass into tissues so its function is limited in scope. IgG molecules are single molecules and can cross epithelial barriers into tissues, or across the placenta. In the human, IgG1 and IgG3 have high affinities for Fc receptors on accessory cells so it can mediate antibody‐dependent cell cytotoxicity (ADCC) and help activate the immune system, these subclasses are also good at complement activation. On the other hand, IgG2 and IgG4 are essentially blocking antibodies since they have very low affinity for Fc receptors and no complement activation. It is worth noting that the mouse classes are not equivalent—IgG3, IgG2b, IgG2c having ADCC capability and IgG1 is the blocking subclass. Another difference between human and mouse is in IgA, where humans have two subclasses and mice only one. IgA is a mucosal antibody and can be secreted across barriers in the gut, breast, lungs, GU tract to block pathogens at mucosal surfaces. The major differences between IgA1 and IgA2 lies in the presence of the drastically extended hinge region of IgA1, thought to improve antigen recognition by increasing affinity with antigen epitopes that are spatially distant, but making it vulnerable to proteases.^37^, ^38^, ^39^ The IgE antibody has received an increasing amount of attention because of its role in hypersensivity responses and allergy in the developed world, although initially thought to have evolved to target parasites (eg, helminths and parasitic arthropods) that are too large to be phagocytosed.^40^, ^41^, ^42^

Class switching can be regulated by multiple factors and pathways, both T‐dependent and T‐independent. As is the case for somatic hypermutation, class switching requires AID, and is most often associated with the germinal center where interaction with T cells via CD40 is critical for the process. Experiments in T cell deficient and CD40 deficient mice have illustrated that germinal center‐independent class switching can also occur, providing the correct stimuli are present. Signaling via Toll Like receptors (TLRs) can complement signaling through the BCR to activate both the non‐canonical and canonical NFkB pathways and initiate class switching.^43^ Similarly, binding of APRIL or BAFF, produced by accessory cells such as neutrophils,^44^ innate lymphoid cells^45^ or fibroblasts,^46^, ^47^ to TACI on the B cell surface will activate the NFkB pathway via MyD88 to cause expression of AID and class switching.^48^ Expression of AID can also be increased by estrogen acting via the HoxC4 AICDA gene activator.^49^

The isotype that a B cell will switch to is affected by the environment and signals that the cell receives. In a T‐dependent response the cytokines produced by T‐helper cells have a critical effect on class switching; IL4 encourages switching to IgG1 and IgE, IL5 and TGFβ encourage switching to IgA, IFNγ encourages IgG3 and IL10 encourages IgG1 and IgG3. There are many other factors which influence the type of class switching. An analysis of the constant region class switch sites in the DNA sequence has revealed many examples of steroid hormone receptor binding sites. Vitamin A helps class switching to IgA and away from IgE, and Vitamin D has also been shown to regulate IgE production.^50^ The discovery of potential nuclear receptor binding sites in the regions of DNA that control class switching raises the possibility that class switching could be directly controlled by vitamins and hormones.^51^ Metabolites such as prostaglandins can also have an effect, PGE2 acting via STAT6 enhances IL4‐mediated class switching to IgE^52^ and can increase IgG1 class switch via cAMP.^53^

The class of an antibody is determined by the constant region gene that follows the VDJ variable region on the immunoglobulin heavy chain gene. In humans, the genetic order of constant region genes in the genome on Chromosome 14 is μ, δ, γ3, γ1, α1, γ2, γ4, ε, and α2. Multiple consecutive switches between different classes and subtypes may occur. Both class switching and somatic hypermutation are related, both occurring after activation by antigen and requiring AID, therefore class switched antibodies will exhibit hypermutated Ig genes. Since mutations accumulate gradually during a response, the temporal events in the life of an activated B cell clone can be ordered by using the level of somatic hypermutation as a molecular clock. Thus, the prevalence and order of class switching can be estimated by analyzing lineages in high throughput Ig repertoire data.^54^, ^55^ The dominant class switching pathway (approximately 85%) is from IgM/D to IgG1 or IgA1 and switching to the downstream classes is usually achieved by sequential events, for example, from IgG1 to IgG2 or IgA1 to IgA2. The “time”, in terms of hypermutation accumulation from one class switched gene to a further downstream one, is less than the “time” taken for IgM/D switching in the first place. More closely related cells are more likely to switch to the same class than more distant ones, in vitro as well as in vivo, possibly as a result of an imprinted state being passed on to progenitors.^54^

## 3 REPERTOIRE ANALYSIS APPROACHES

Techniques that amplify and sequence the repertoire have been collectively referred to as Rep‐Seq.^56^ The initiating step in B cell repertoire studies was the identification of a full suite of PCR primers that could amplify all expressed heavy chain variable regions in a consensus PCR.^57^ Early Ig repertoire analysis used PCR primers that bound in the Variable and Joining regions of the rearranged Ig genes to prepare the amplicon libraries for sequencing. While this had the advantage of being a robust method it did not produce data on the antibody class unless the cells had been sorted using surface markers prior to library generation. It also potentially biased the measurements of J region usage and was open to the risk of V region bias due to faulty primers by virtue of the fact that the V region primers were a mix of family‐specific primers. While these early sequencing technologies were invaluable for the discovery of new cell populations, they often relied on expensive and time‐consuming cloning that did not capture the full repertoire; due to the single channel capabilities of Sanger Sequencing.^20^, ^22^, ^29^, ^58^

Advances in Rep‐Seq in terms of primer design, coupled with next‐generation sequencing, enabled the full repertoire to be explored with the only drawbacks being difficulty amplifying rare heavy chains, PCR and sequencing bias, and amplification of IgG which is consistently less efficient than other heavy and light chains. A further step forward came with the use of template switch enzymes and 5′ RACE, as has been frequently used in T cell biology.^59^, ^60^, ^61^, ^62^, ^63^, ^64^, ^65^ The 5′ RACE method has an advantage over consensus immunoglobulin PCR because it only requires priming in the constant region and adds a primer landing site in the 5′ with the addition of a template switch oligo (TSO). The TSO anchors to the non‐template strand during reverse transcription by means of oligo(rG) allowing the enzyme to switch templates onto the TSO from the immunoglobulin mRNA.^63^, ^66^, ^67^, ^68^ The 5′ RACE technique therefore reduces PCR bias but may result in less efficient transcript capture and reduced repertoire diversity over other amplification methods. Another advantage of 5′ RACE is the further inclusion of unique molecular identifiers (UMIs), random strings of nucleotides that could be added to a primer making each primer sequence unique, allowing bioinformatics resolution of the PCR bias problem (see below). Bioinformatic tools for the reconstruction of the repertoire from mRNA‐seq data are now also becoming available.^69^

The ability to distinguish between subclasses would not, however, be possible without major advances in high throughput sequencing. Of the early next‐gen platforms 454 was typically favored^70^, ^71^, ^72^, ^73^ over early Illumina or SOLiD in antibody analysis because of capacity to produce longer reads that could also allow class/subclass determination. Methods using paired end Illumina sequencing have advanced, however, allowing the capture of longer reads and sequencing of the full variable region and subclass isotyping with certain 2x 300 bp paired end sequencing methods.^74^ While Illumina offers unprecedented read counts, reconstructing libraries of antibody sequences, which can be in excess of 900 bp if determining subclass, becomes a bioinformatics conundrum, although there are now a large range of tools to facilitate this.^75^, ^76^, ^77^, ^78^ Paired end data can also be limited in ability to distinguish some somatic variants.^79^ As such, the Pacific Biosciences (PacBio) RSII system which offers reads lengths of 10 000 bp on average has become increasingly attractive for specialized applications^80^ despite its comparatively poor reads per run and high cost (see Table 1). The use of barcodes, a string of known nucleotides added to individual samples by using multiple specifically produced primers, allows simple multiplexing on higher cost sequencing platforms but is currently still expensive. We expect that advances in the PacBio read numbers will continue to improve, as has been the case with the release of the Sequel platform offering a ten‐fold increase in read per run over the RSII, while Illumina technology will remain unparalleled in terms of reads per run, but is plateauing on read length improvements. The use of one platform over another in the short term will therefore largely depend on what is required by the researcher (see Table 1).

**Table 1:** Comparison of next generation sequencing methods

	Illumina (300 bp paired end)	Pacific biosciences RSII (per SMART cell)	454 (GS‐FLX Titanium)
Maximum read length	2 × 300 bp	>60 000 bp (10 000 bp average)	700‐800 bp
Reads per run	44‐50 million (Minimum)	55 000^a^ per SMART cell	~1 million
Output	13.2‐15 Gb per run	1‐2 Gb per day	0.7 Gb
Bioinformatics analysis	Some assembly required	Simple	Simple
Ig Class	Generally limited to class only	Subclass possible	Subclass possible
QC issues		2 μg of amplicons required	
Time of run	~65 h	~6 h per SMRT cell	~24 h
Cost^b^	US$1400	US$400 per cell	US$6000
Quality^c^	Q20‐Q30	Q50	Q30

a The newer, but less available, Sequel by Pacific Biosciences is capable of producing ~330 000 reads but at nearly double the cost per cell.

b Costs have been based on a single website (allseq) to avoid provider differences and is based on running at cost. NB the PacBio RSII will take up to 16 SMART chips per run and therefore scales with cells used.

c Quality scores are based on the base calling accuracy of a run. A Q20 has a probability of calling 1 incorrect base in 100 (99% accuracy), Q30 = 1 incorrect base in 1000 (99.9% accuracy), Q40 = 1 incorrect base in 10 000 (99.99% accuracy) ect.

**Figure 2:**
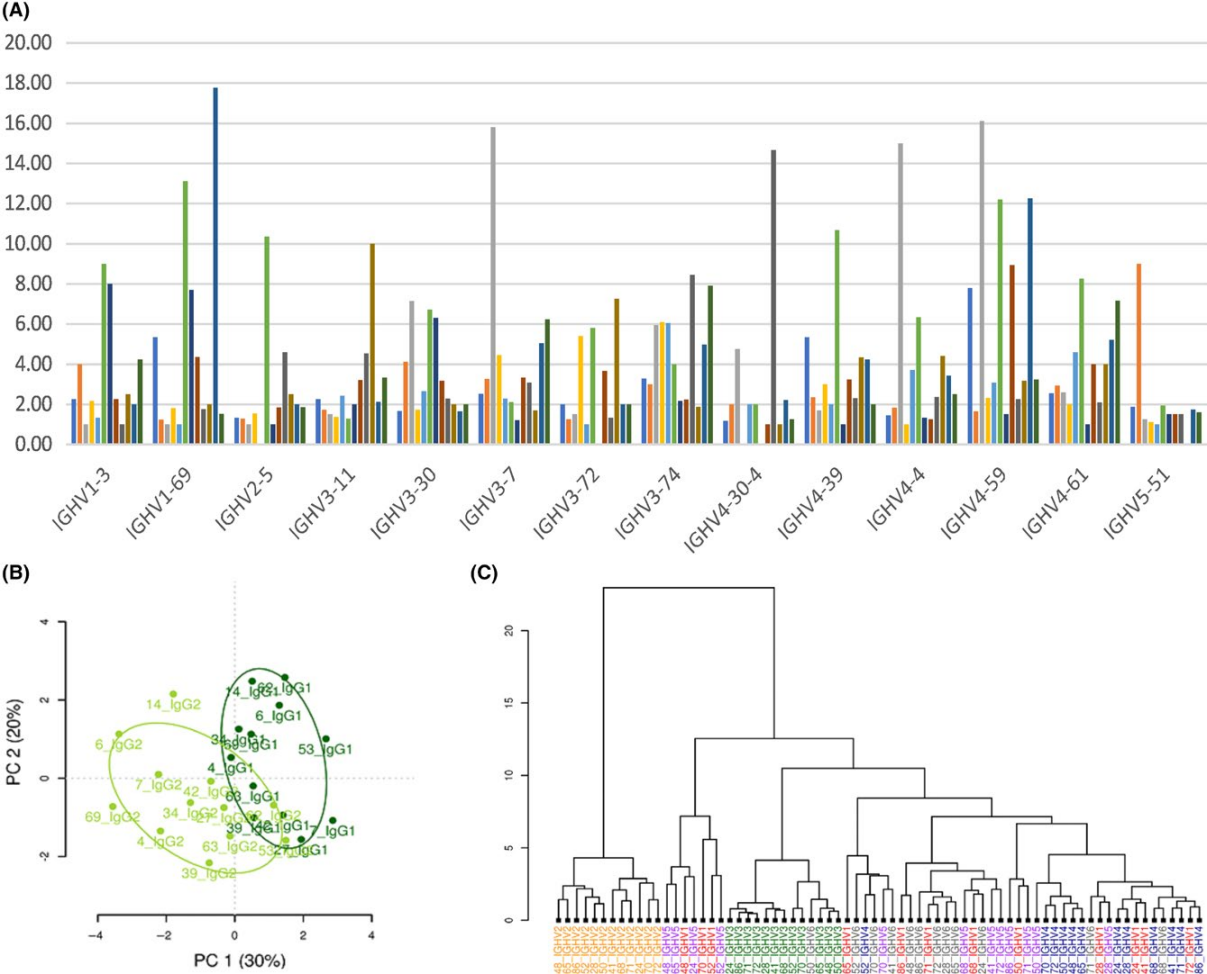
Ig gene repertoire variation between individuals, classes of antibody, and IGHV gene families. (a) Individual variability in a human vaccine response. Average clonality of selected IGHV genes in the repertoire of 12 individuals (each is color coded) at day 7 after challenge with influenza and pneumococcal vaccines.^156^ Average clonality is the number of sequences divided by the number of clonal families for each individual genes. Average clonality of 1 indicates lack of clonal expansion. (b) PCA analysis of CDR3 physicochemical properties, as defined by kidera factors, showing the difference between Ig genes of IgG1 vs IgG2 subclasses. Data from Martin et al^73^ (c) Segregation of IGHV family genes by CDR‐H3 physicochemical properties. Minkowsky distance clustering by Brepertoire^146^ on IgM sequences from B cells in early development in 12 different individuals.^76^ Each sample is a separate individual. IGHV genes color coded: Yellow; IGHV2, Red;IGHV1, Green; IGHV3, Blue; IGHV4, Violet; IGHV5, Gray; IGHV6

With these advances in Rep‐Seq, long read sequencing technologies and with 3′ PCR primers sufficiently far down the constant region, the distinction between subclasses has enabled a full investigation of antibody class in the repertoires. This is important, as we have shown that the repertoire can vary quite substantially by class of antibody. While IgG1 and IgG3 seem to share repertoire characteristics, IgM cells and IgG2 can vary substantially, particularly in younger adults.^81^ In older adults, the selection events shaping the repertoire seem to change.^8^ In human, the main variations in IGHV gene usage seem to be in the relative use of IGHV1 and IGHV3 family genes,^81^ although CDR3 character can also distinguish between populations (Figure 2b). The reasons for this are not clear; one possible hypothesis is that there is a peripheral tolerance mechanism preventing expansion of potentially dangerous Ig genes, ie, genes with potential to do self‐harm. Potentially harmful Ig genes still exist in the repertoire, as there is a trade‐off between tolerance safety and having sufficient capability to detect diverse pathogens. The cells carrying these genes are not allowed to expand without licencing by help from other cells. So that in the classical T‐dependent B cell response, producing IgG1 and IgG3 antibodies, the potentially harmful genes can survive, and in a T‐independent B cell response, producing IgM memory and IgG2 antibodies, they cannot, thus skewing the repertoire. In this example we suggest that IGHV1 family genes have more potential for harm than IGHV3 genes, thus explaining the increased IGHV1 gene use in IgG1 antibodies and decreased use in IgM memory or IgG2 responses. Perhaps not surprisingly, B cells at different stages of their developmental pathway can also have different repertoires. We have shown differences in the periphery between transitional cells (IgD+IgM+CD27−), naïve cells (IgD+IgM+CD27−), IgM memory cells (IgD+IgM+CD27+), classical switched memory cells (CD27+IgD−) and CD27− switched memory cells (IgD−CD27−)^82^ and others have extended this to show plasmocyte differences.^83^ We have also shown repertoire differences as B cells progress through bone marrow development and central tolerance.^84^ These studies all serve to reinforce the view that repertoire studies should be conducted on sorted cells, be class and subclass‐specific and the subjects should be age matched as well as possible.

The most recent advances in Rep‐Seq have come with the use of single cell technologies which allow the full antibody structure, both the heavy and light chain from a single cell, to be uncovered. These technologies often also have the capacity to produce single cell transcriptomic data (scRNA‐seq), the estimated prices for some of the more popular methods are included in Table 2 and see also Ziegenhain et al^85^ for a more comprehensive list on scRNA‐seq. The first of these technologies to be applied extensively used FACS or a microfluidic devise to deposit single cells into a well allowing for Ig specific RT‐PCR of a single cell^86^, ^87^, ^88^, ^89^, ^90^; the major drawback being low throughput. These techniques have, however, rapidly been succeeded with microfluidic technologies which have massively increased throughput, allowing thousands of heavy and light chains to be bound together and sequenced as a single entity. Microfluidic equipment for this “DropSeq” method has been bespoke in a number of labs, although there is now a commercially available system from Dolomite Bio suitable for this application. These single‐cell microfluidic methods rely on a PCR that is simple in concept, joining the heavy and light chain transcripts by over‐lap extension, but difficult in practice given the large number of primers in a single approximately 65 pico‐liter emulsion droplet.^79^, ^80^, ^91^ The joining of both the heavy and the light chain resulting in an amplicon that may be in excess of 1000 bp has made it a prime candidate for long read sequencing technologies. As yet, however, this technology has not been adapted to allow isotyping of subclasses.^79^, ^80^ Bioinformatic methods that use scRNA‐seq data may also be used to reconstruct the joint heavy/light chain repertoire coupled with the full transcriptome.^92^, ^93^ In 2017 10x Genomics produced chemistry kits for their Chromium machine which are capable of producing barcoded libraries for sequencing that can be separately enriched for BCR or TCR data. To date, however, we have not seen any publications that have implemented this. We believe that these new joint heavy‐light chain technologies will form the basis of repertoire analysis in the future, as was the case with class and subclass isotyping, because of the additional structural and full variable region data that can be attained.

**Table 2:** The costs of running some of the more prominent single‐cell technologies. Note that prices are estimates and may vary as a result of different suppliers, exchange rates and prices scalable on quantity purchased. None of these costs include sequencing, see Table 1

	scRNA‐Seq			Paired heavy‐light chain	
	Drop‐Seq	10x genomics^c^	Smart‐seq	Overlap‐extension	10x Genomics^c^
Equipment cost	US$50 000‐65 000^b^	US$75 000	N/A^a^	US$55 000^b^	US$75 000
Per run cost	US$500‐700	US$1288	US$1000	US$400‐500	US$1288
Cells per run	~10 000	100‐100 000	96‐384	100 000‐150 000	100‐100 000
Estimated time to process a run (h)	24‐48	24‐48	48‐72	24‐48	24‐48
Capture efficiency	5‐10%	65%	100%	>90%	65%

a Although Smart‐seq does not require any specialized equipment it does require the ability to sort cells into 96 or 384 well plates.

b This cost is based on an ‘off the shelf’ model although methods exist for self‐assembly. For Drop‐Seq and Ig pairing by overlap extension we have used Dolomite Bio as our reference. In this case as well, buying the equipment for one method will reduce the equipment purchase price for the other as parts are interchangeable.

c The 10X system uses the same machine for both methods. Note that the system will also perform both scSeq and paired heavy light chain from the same sample for US$65 more and TCR on top of that at an additional US$65.

## 4 CLONALITY ANALYSIS

Given the available genes, and the probabilities of nucleotide excision/addition, the CDR‐H3 region of heavy chain gene rearrangements is highly diverse, producing unique sequences at each rearrangement event. There are some rare instances, where the CDR‐H3 is very small such that the probabilities weigh in favor of seeing the same CDR‐H3 in two different rearrangement events,^94^ but in general the CDR‐H3 can be used as a fingerprint for a particular B cell and its progeny and one would not expect to see two different B cells with the same CDR‐H3 in a small sample unless they were related. Clustering immunoglobulin sequences into “clones” allows studies of B cell relationships between different samples and can facilitate the study of repertoire both as a whole, and also looking at the background diversity without the effects of clonal expansion.

### 4.1 Dissemination

Matching IGH genes with the same CDR‐H3 in different areas of tissue can be used to show the dissemination of effector cells between different sites and we first used this in microdissected areas of tissue to illustrate that lamina propria plasma cells are highly mutated and originate in Peyer's patches of the gut.^22^, ^95^ With high throughput sequencing technologies, it has been possible to undertake such dissemination analysis on a much larger scale and to show that there is a certain amount of compartmentalization between mucosal vs systemic tissues in the distribution of B cells.^96^ Analysis of clonality on a large scale requires considerable computational resource, and there have been various methods employed over the years. The data need to be analyzed at the nucleotide level to give sufficient discriminatory power and to cope with the complications brought about by hypermutation and sequencing error. These complications also frustrate a definitive clustering of Ig gene sequences into “clones” so all experiments should be comparative using exactly the same methods. We have used a levenstein distance, as opposed to a hamming distance, to build hierarchical clustering dendrograms in order to reduce error introduced by sequence indel errors.^84^ This is important where HTS sequencing platforms are prone to homopolymer tract errors as CDR‐H3 regions often have larger homopolymer tracts. We use an empirically determined cut off value to split the sequences into clonal groups which errs on the side of inclusivity. Since hypermutation levels will always confound this analysis it is impossible to get 100% specificity and sensitivity in the clonal allocation, but it is easier to split an incorrectly clustered clone upon closer inspection than it is to know about potential missing sequences. A recent paper concluded that single linkage hierarchical clustering with Hamming distance has high performance, with specificity, sensitivity, and positive predictive value all over 99% in their test data.^97^ More complex clustering algorithms can be employed, such as multi‐hidden Markov models^98^ which can give different results to hierarchical clustering methods. Therefore, it is important to check the clustering methods employed if one wants to compare results from different studies. To this end, recent and ongoing work by the Adaptive Immune Receptor Repertoire (AIRR) consortium to set international standards for data sharing and tools repositories will enable more comparison of data from multiple sources in the future.^99^

### 4.2 Clonal expansion

A key factor in assessing the immune response is to identify the extent of in vivo clonal expansion as the B cells with receptors specific to the challenge are positively selected. This can give us information as to whether the response is focussed, with a few very large expansions, or broad, with many smaller expansions. It can tell us the health of the baseline repertoire in terms of diversity, such as seeing more clonal expansions in the absence of challenge, or less timely contraction of the repertoire after challenge, in older people.^100^

An important caveat to note with all clonality analysis of HTS data is that the results can easily be skewed by the methods employed. Firstly, the creation of libraries of genes is done by polymerase chain reaction (PCR) amplification and so over‐sequencing of the library will skew the results to reflect PCR expansion rather than in vivo expansion of the Ig genes. Some of the earlier HTS data was produced in this way.^101^ This can be overcome using methods that incorporate UMIs at the reverse transcription step so that only one copy of each mRNA molecule is counted.^102^ This method could also be used to align copies of the same sequence to identify and remove sequencing errors in high read methods. In lower read methods, typically 60 000 sequences per experiment for PacBio long reads, for example, we have found that overcounting of sequences is not a problem (data not shown). This would only be the case if the input quantity of mRNA was sufficient and, since maintaining mRNA quality is one of the highest risks in these experiments, we would advocate the use of UMIs for all future data sets. In addition, the use of mRNA as a starting point has its own issues in that not every cell will have exactly the same number of mRNA molecules, so an assumption that one Ig gene sequence represents one cell in vivo is incorrect. For most B cells there is correlation with the number of Ig gene sequences and cells, but plasma cells have 100 times more mRNA for Ig genes than other B cells. Sequencing of genomic DNA would negate this issue, but then UMIs could not be added by RACE methods. More importantly, we would not be able to find information on the class of antibody under investigation. It is our recommendation that Ig gene repertoires be prepared from mRNA isolated from presorted B cells, adding UMIs and, using 3′ PCR primers in the constant region that allow later discrimination between antibody subclasses.

Given the technological capability of producing monoclonal antibodies for therapeutics there are many instances where we would like to know the sequence(s) for the antibody/antibodies responding to a particular challenge. It has been assumed that a B cell clone that is most expanded in response to challenge would be the most useful in protecting the host from the challenge. Indeed, there are several reports where the predominant clones in a response have been shown to bind the antigen.^103^ In mice these experiments have been particularly successful.^104^ However, the assumption of largest clone providing best protection may be too simple, and many different immunoglobulin genes can respond to a single challenge. Human studies have the additional challenge that only a sampled snapshot of the repertoire can be examined. One of the earliest reports of heavy/light chain repertoire in human tetanus vaccine response illustrated the breadth of responding genes across the repertoire.^86^ While different people can share similarities of repertoire, there are aspects of an individual repertoire that are unique to that person^105^ and they may not always expand the same Ig genes in response to challenge. Figure 2a shows the broad nature of an expansion response, differing between individuals, for the same vaccine challenge. A diverse response is beneficial, a comparison of Avian flu survivors vs non‐survivors found one of the chief differences was the diversity of the B cell repertoire, where increased diversity correlated with survival.^106^ Repeated sampling of the repertoire over time can be helpful in identifying potentially protective antibodies^107^ and convergence of repertoires between different people toward similar Ig genotypes has been shown, for example, in response to influenza,^108^ meningococcal^109^, and Dengue^110^ vaccines. However, finding a convergent signature for equivalent challenging antigen preparations may not always be possible, even when temporal data for the response is available.^107^ Comparison of predicted sequences from the whole repertoire with sequences obtained after sorting B cells labeled with the specific antigen can help to develop models for in silico prediction of antigen‐specific sequences in a repertoire.^111^ We do need to bear in mind that a sampling of blood B cells for sequence repertoire is not the same as sampling the antibodies produced in response to challenge.^112^ The latter are produced by plasma cells in the bone marrow and the former are more diverse. In addition, we cannot always assume that a large clonal expansion of IgG would indicate best protection. Other classes of antibody have been shown to be important, such as IgM in Ebola,^113^ which may be less focussed in their clonal expansion response. In our laboratory, preliminary experiments using ribosome display to capture antigen‐specific sequences do find sequences that we see in the whole repertoire, but not in the largest clonal expansions and often are isotypes other than IgG.

### 4.3 Clonal evolution

Examination of clustered data on an individual clone level can provide information about the evolution of a B cell clone as the Ig genes acquire mutations in the immune response. It is important to know whether an ongoing expansion of cells is just that, expanding exactly the same immunoglobulin gene, or whether there is also ongoing mutation involved—which would imply the involvement of a more complex germinal center reaction and affinity maturation. Determining the relative position of cells from different phenotypical subsets within a lineage tree may also be able to provide information as to the order of lineage relationships. We have used manually curated lineage trees to show changes in germinal center selection with age, relationships between different types of memory B cells and ongoing diversification in MALT lymphoma.^24^, ^29^, ^114^ Transferring these more in‐depth analyses to high throughput methods is dependent on the accuracy of sequence information, and there is a sense of reluctance in the field to take clear biological inferences from what may not be the most precise data. HTS methods that incorporate UMIs and that provide multiple reads of the same unique sequence may be able to provide data which would overcome this reluctance and it may even be possible to correct sequencing data without the aid of UIDs with the appropriate algorithm such as IgReC.^78^ In addition, there are computational methods available for the construction of lineage trees.^115^, ^116^ We also need to recognize that allelic variants may exist in the population that may not be represented in germline gene databases and therefore some “mutations” from germline may be miscalled. These could potentially skew hypermutation data from different patients and there are now methods for predicting germline genes by inference from high throughput data which can help overcome this issue.^117^, ^118^, ^119^, ^120^

The earliest analyses of antibody lineage trees employed graph theory to extract metrics with respect to the shape of the trees and analyze how these correlated with biological parameters.^24^, ^121^, ^122^, ^123^, ^124^ Later methods are reviewed elsewhere.^125^ The shapes of the lineage trees give important information about the history of the B cell clone, for example the extent of selection acting on a B cell clone can be reflected in the shape.^121^, ^126^ A preponderance of trees with long trunks in a population would indicate that many clones started from pre‐mutated (memory) B cells as compared to lineages which branch close to the origin, which would be more likely to have started as a naïve B cell. In mutation analysis for the purposes of inferring information about selection and mutational targeting, hypermutation events should not be counted by counting every mutation on every sequence—but rather in the context of lineage trees so that each mutational event is only counted once. One crude way of doing this is to randomly pick one sequence per clone for analysis. More sophisticated methods analyze each mutation as it occurs in the lineage of the antibody. This captures all the mutational diversity within the clone and would also be more accurate with respect to positional effects, since each mutation position would be considered in the context of the flanking sequences at the time the mutation occurred rather than the germline sequence. The most recent tools for lineage analysis use modern statistical molecular evolution methods on nucleic acid sequences,^36^ or on amino acid sequences.^127^

## 5 GENE USE ANALYSIS

Comparison of the frequency of use of different immunoglobulin genes between different samples is a useful biomarker for biological skewing of the lymphocyte repertoire. Some individual genes have been identified as being associated with human disease. *IGHV5‐51* is associated with Celiac disease.^128^ *IGHV4‐34* has often been associated with autoimmune disease and chronic lymphocytic leukemia.^129^, ^130^, ^131^ *IGHV4‐34* has been shown to bind citrullinated protein antigen in rheumatoid arthritis,^130^ but it also has a unique framework 1 region that can bind to human red blood cell antigens I and i when in its germline form,^132^ these antigens can therefore be considered to be superantigens. It is one of few antibodies that has an N‐glycosylation site in the germline IGHV region, and it has been hypothesized that the potential autoreactive binding potentials can be modified by changing glycosylation in a germinal center reaction.^133^ Another superantigen is Staphalococcus protein A, which can bind regions in framework 3 of IGHV3 family genes,^134^ while some IGHV3 genes have been associated with disease, such as *IGHV3‐21* in CLL,^135^ individual *IGHV3* gene expansions are less commonly found. IGHV1 genes have consistently been implicated in disease, with *IGHV1‐69* featuring prominently in CLL in the western world. There may be geographical/ethnic variation, with *IGHV2‐5* and *IGHV1‐2* also featuring in CLL in India,^136^ and lower levels of *IGHV1‐69* in Japan,^137^ but in Europe up to 30% of CLL are of *IGHV1‐69*‐carrying cell origin.^138^ *IGHV1* genes are also very important in protection against viral infections, *IGHV1‐69* genes having been associated with influenza, hepatitis B and hepatitis c, HIV.^139^, ^140^, ^141^ The *IGHV1‐46* gene has been shown to bind both rotavirus antigen VP6 and autoantigen desmoglein in pemphigus disease.^142^ So, it seems that the trade‐off between risk of autoimmunity vs protection from viral infection is particularly finely balanced for *IGHV1* genes. In spite of these examples, in well over a decade of studies on human repertoire in health and disease, it is somewhat surprising that there have been so few *IGHV* gene associations made with antigen specificities. This may be due to confounding by interindividual variation. It is difficult to say what a normal unselected repertoire would be, since bone marrow samples are difficult to obtain and cell separation methods not adequate to distinguish the initial light chain rearrangements from the results of receptor editing. There are some excellent attempts at modeling the potential baseline,^3^ but more data to test these models would be required for them to become of general use. Looking for individual genes may not be the only biomarker of relevance, and modern bioinformatics with B cell repertoire sequencing has been used in the last few years to identify different biomarkers associated with diseases such as multiple sclerosis.^143^ One area we believe to be of particular significance is the CDR3 properties of the sequences and the structural information of the antibody when it is available.

## 6 CDR3 CHARACTERISTICS

The question of which part of the antibody is the most important for antigen binding is an interesting one. As mentioned above, the CDR3 region is the most variable part of the antibody by virtue of the contributions from the different genes at the junction and the imprecise nature of the gene rearrangement process. Mice restricted to a single Variable region gene have shown that they are capable of eliciting high affinity responses to various protein and hapten challenges, which is evidence to support the idea that CDRH3 is the most important sequence conferring specificity of the antibody.^144^ They did find that their arbitrarily chosen V region did not support binding to T‐independent polysaccharide antigens, so there is reason to believe that CDR1 and 2, and perhaps other aspects of the sequence are also important for certain classes of antigen. Other evidence suggests that V gene use makes a significant difference to antigen recognition. Contact residues may not always be part of the CDR^145^ and the same CDRH3 on different heavy/light chain backgrounds can take on different structures.^146^ As a result of the complexities of protein folding behavior, selection of mutations for affinity may not be directly related to contact residues.^147^ We looked for any biases in CDR3 properties between different IGHV family genes in our data. While most IGHV genes did not appear to affect the CDR3, use of IGHV2 family genes showed a skewing in CDR3 properties compared to the rest of the repertoire, indicating IGHV2 has an effect on CDR3 structure that in turn affects antigen binding sufficiently to affect repertoire selection (Figure 2c). That said, *IGHV2* family genes are a very small fraction of the repertoire as a whole, so while it is worth bearing in mind when interpreting CDR3 repertoire information it would only be of concern if the IGHV2 component were altered for any reason.

Much work on the effects of changing CDR3 sequence on antibody specificity has been done in mice^148^ and only since the advent of spectratyping and high throughput sequencing have we done any serious analysis of human CDR‐H3. One of the most consistent changes in repertoire we see is the change in CDR‐H3 length in B cell development. During an immune response to vaccine the whole blood repertoire shifts toward a smaller CDR‐H3, across IgG, IgA and IgM, at the peak of the plasmablast response before returning to baseline by day 28.^149^ During early B cell development of IgM, between preB cells to Naïve B cells, there is also a significant decrease in CDR‐H3 size.^84^, ^150^ The size of CDR3 is determined partially by IGH gene use, and partially by factors involved in gene rearrangement at the junctions—particularly the activity of Terminal deoxynucleotidyl transferase (TdT) adding random nucleotides to the junction. There may be interindividual variation in TdT activity since, in young adults, the distribution of CDR3 size at baseline and day 28 is similar within the individual, but different between individuals.^151^ Similarly, the level of N nucleotide addition in early B cell development is consistent between heavy, kappa and lambda chains within individuals, but differs between individuals.^152^ Given the apparent importance of CDR3 size to an antibody response^82^, ^149^ and to central tolerance^84^, ^150^ these interindividual differences may warrant closer inspection in studies on immune disease, vaccination and infection as they may be biomarkers of response or autoimmunity.

The physicochemical characteristics of the CDR3 are also important, not only from the point of view of how they affect protein folding, and therefore the shape space of the binding site, but with respect to their ability to interact with other molecules. For example, folding of the CDR‐H3 can be affected significantly by the presence of pairs of cysteines, which can form disulphide bonds.^147^ We found that there is some selection against the use of cysteines in central tolerance; the percentage of sequences without any cysteines increases from 85% to 91% between preB and naïve B cells. Although it is difficult to infer an antibody's specificity based on its amino acid sequence, it has been observed that the CDR‐H3 regions of antibodies in the bone marrow are on average longer, and more hydrophobic than those in the peripheral blood^84,1151,152^, indicating that these CDR‐H3 characteristics are selected against during central tolerance. The charge at the binding site is also critical, the prevalence of positively charged arginines in the CDR3 has been associated with binding to (negatively charged) DNA in some antibodies and in SLE^153^, ^154^ and to phospholipid antigens.^155^ We have shown that the number of arginines, and the other charged amino acids histidine and lysine, can vary significantly between different B cell populations with an overall increase in moving from the naïve to the memory populations,^82^ perhaps indicating that charged interactions are important for binding to exogenous antigen. The other key property of the antibody binding site is hydrophobicity. It has been suggested that hydrophobic patches are associated with polyspecificity of binding and it has been shown that antibodies with hydrophobic patches in their CDR3 are prone to aggregation. This can be abrogated, without loss of specificity, by changing amino acids at the edge of the CDR3.^156^ In addition to decreasing hydrophobicity through early B cell development,^84^, ^150^, ^157^ we have seen a decreased hydrophobicity in memory cells compared to naïve cells,^82^ which would be consistent with tolerance selection during an immune response.

There are actually hundreds of different metrics to assess the physicochemical properties of a protein or peptide, many of which overlap in function. Kidera et al^158^ determined a set of 10 orthogonal factors (KR 1‐10) which could capture a broad range of information. We have incorporated the calculation of these into our BRepertoire tools^159^ and found that they can be used in PCA analysis or Minkowsky distance clustering to distinguish between different samples, such as B cells from different developmental stages.^84^ In addition to hydrophobicity and charge, we can see differences in other properties. For example, in the comparison between IGHV2 genes and the rest of the repertoire (Figure 2c) we found significant changes in KF2:Side chain size, KF5:Double bend preference, KF6:Partial specific volume, and KF7:Flat extended preference.

## 7 ANTIBODY STRUCTURE

Given the differences in CDR sequence characteristics between antibodies it is easy to see that the information of real relevance to design of effective antibodies lies in the structure encoded by that sequence. The major hurdle to date has been that immunoglobulin repertoires have either been single chain only, or have been too short to have the full sequence of both chains. Assuming that the single cell and long read technologies will be able to correct this in the near future, then the next challenge will be modeling the protein structure. The steps involved in modeling are reviewed in detail elsewhere,^151^ and the challenges are mainly with the CDR3 regions for which suitable templates are not always available in the protein data bank (PDB). We have produced some structures for antibodies that are polyreactive, showing that their long CDR‐H3 loops appear to project out of the antigen binding site, but the longer the CDR‐H3 then the more likely the antibody would have a flexible conformation and this work is still in its preliminary stages.^160^ Others have usefully employed modeling techniques to investigate the maturation of anti‐HIV and anti‐influenza antibodies.^161^ The pipeline for our modeling to date involves making multiple models initially and picking the best one before performing multiple simulations of conformation, using tCONCORD to give an ensemble that can be analyzed.^160^ Although this rigorous treatment gives us confidence in the predicted structures, it is computationally quite expensive and difficult to apply in high throughput. A recent paper that used the RosettaAntibody 3.0 antibody modeling protocol^162^ estimated that modeling of 2000 sequences took approximately 570 000 CPU hours^163^ so clearly there are challenges in the development of tools for structural calculations at a scale to match the available repertoire information. Of the large number of different tools currently available it seems that ABodyBuilder is the speediest, at 30 seconds per structure, which is around 567 CPU hours per thousand sequences.^151^, ^164^, ^165^

In addition to protein folding, the glycosylation status of antibodies is important. Not many immunoglobulin genes have N‐linked glycosylation sites in their variable regions in germline configuration (*IGHV4‐34, IGHV1‐8, IGHV5‐a*), but it is possible to gain these sites through somatic hypermutation.^133^ High throughput repertoire studies show that some genes are more likely to acquire an N‐glycosylation sequon than others, for example, *IGHV3‐23* and *IGHV6‐1* 133 and sequons are more often found in or near the CDRs where they are more likely to affect antigen binding.^166^ While in most instances the lack of glycosylation on selected antibodies would indicate that the glycans block or reduce binding, there are a few instances of N‐glycosylation conferring increased antigen specificity.^166^, ^167^

## 8 SUMMARY

There are many areas of biology and medicine where the information available from repertoire data can provide valuable insight. With the increasing importance of biologics as therapeutics, repertoire studies also have a valuable place in the discovery and design of antibodies and chimeric antigen receptors. The study of such large numbers of sequences, with all the complexities that they entail, has resulted in an interdisciplinary field that encompasses immunologists, physicists, computational biologists and mathematical modelers as well as providing a substantial collection of methods and tools. The immediate future directions are to encourage order and standards with respect to tools and data repositories, while at the same time improving existing biological and computational methods to address the challenge of producing accurate paired chain repertoires with tractable high scale structural modeling methods.

## CONFLICT OF INTEREST

Authors have no conflict of interest.
